# Comparing ESC and iPSC—Based Models for Human Genetic Disorders

**DOI:** 10.3390/jcm3041146

**Published:** 2014-10-24

**Authors:** Tomer Halevy, Achia Urbach

**Affiliations:** 1 Stem Cell Unit, Department of Genetics, Institute of Life Sciences, The Hebrew University of Jerusalem, Jerusalem 91904, Israel; E-Mail: tomerhalevy@gmail.com; 2 Mina and Everard Goodman Faculty of Life Sciences, Bar-Ilan University, Ramat Gan 5290002, Israel

**Keywords:** embryonic stem cells (ESCs), induced pluripotent stem cells (iPSCs), disease modeling

## Abstract

Traditionally, human disorders were studied using animal models or somatic cells taken from patients. Such studies enabled the analysis of the molecular mechanisms of numerous disorders, and led to the discovery of new treatments. Yet, these systems are limited or even irrelevant in modeling multiple genetic diseases. The isolation of human embryonic stem cells (ESCs) from diseased blastocysts, the derivation of induced pluripotent stem cells (iPSCs) from patients’ somatic cells, and the new technologies for genome editing of pluripotent stem cells have opened a new window of opportunities in the field of disease modeling, and enabled studying diseases that couldn’t be modeled in the past. Importantly, despite the high similarity between ESCs and iPSCs, there are several fundamental differences between these cells, which have important implications regarding disease modeling. In this review we compare ESC-based models to iPSC-based models, and highlight the advantages and disadvantages of each system. We further suggest a roadmap for how to choose the optimal strategy to model each specific disorder.

## 1. Introduction

**Figure 1:**
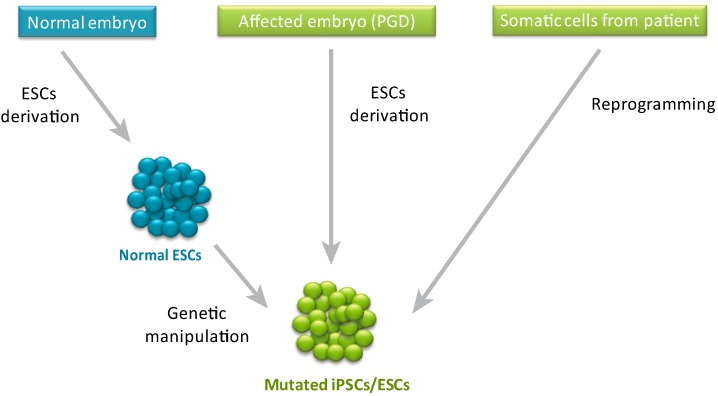
Human pluripotent stem cell-based models for genetic disorders can be generated by different techniques. Mutated human pluripotent stem cells can be derived by genetic manipulation of normal pluripotent stem cells, from affected embryos (identified by PGD), or from adult patients (by reprogramming of somatic cells).

Pluripotent stem cells have an unlimited self-renewal capacity and can differentiate into virtually any adult cell type [1] and even some extra-embryonic tissues [2,3]. These features make human pluripotent stem cells (hPSCs) a useful tool for disease modeling, which overcomes limitations observed in animal and adult human cellular models. While the use of animal models proved to be extremely valuable and successful in many cases [4], there are numerous diseases, such as Lesch-Nyhan syndrome [5], Turner syndrome [6] and Fragile X syndrome [7], that cannot be studied using animal models due to species-specific differences. The use of mature cells from patients can solve the species-specificity issue but this strategy is limited by the fact that it enables studying only a few types of cells at a specific developmental stage, and in many cases requires also transformation of the cells to enable their proliferation in culture. By contrast, due to their unique properties, hPSCs enable exploration of different types of cells, to study the effect of a specific mutation on differentiation or development and can proliferate *in vitro* without additional transformation. Indeed, since the generation of the first human embryonic stem cells (ESCs) based model (a model for Lesch-Nyhan syndrome by targeting of the *HPRT* gene in human ESCs) [5] dozens of disease models were generated by reprogramming of somatic cells from patients [1], by derivation of mutant ESCs from affected embryos diagnosed by *pre*-implantation genetic diagnosis (PGD) or by genetic manipulation of normal ESCs [8] (see Figure 1). While some models were used as a “proof of concept” to demonstrate that hPSCs can be derived from a wide range of disorders [9,10,11] or to show the feasibility of the mutant pluripotent cells to be used as a disease model [12], other models were further used to obtain novel mechanistic or physiological insights regarding the disorders. One example is a model for Amyotrophic Lateral Sclerosis (ALS) by Kiskinis *et al.* [13].

The general differences between ESCs and induced pluripotent stem cells (iPSCs) and the utilization of hPSCs for disease modeling has been discussed extensively in the literature [1,14,15,16]. In this review we will focus on the differences between ESC-based models and iPSC-based models, and discuss the effect of genome editing technologies on the field of disease modeling.

## 2. ESCs *vs.* iPSCs in Disease Modeling

Theoretically, a given disorder can be equally modeled by iPSCs and by ESCs, as both are pluripotent stem cells. However, several reasons have made iPSCs derived from patients the system of choice:

(1) The use of normal human ESCs to model a genetic disorder requires genetic manipulation to induce the specific mutation that one would like to study. The way to obtain a mutation that will be identical to the natural occurring mutation, seen in patients, is by genome editing. However, the efficiency of genome editing in human ESCs, before the establishment of gene targeting technologies as discussed below, was extremely low (especially in cases where a homozygous mutation was required) [17] and derivation of iPSCs that already contain the specific mutation obviates the needs for this inefficient process.
(2) While the above mentioned limitation can be overcome by derivation of mutant ESCs from affected embryos identified by Preimplantation Genetic Diagnosis (PGD), this procedure is limited to a small number of diseases in which PGD is normally preformed, and can be done only in labs that are associated with *in vitro* fertilization (IVF) units.
(3) By contrast to iPSCs from affected individuals, in the case of ESCs based models, the correlation between the genotype and the phenotype is not obvious, and the penetrance of the mutation might be low as a results of specific “protective” genetic background [18].
(4) Lastly, in some countries the use of human ESCs is limited or banned due to ethical and religious concerns regarding the use of human embryos for research purposes as was discussed by others [19,20].

**Figure 2:**
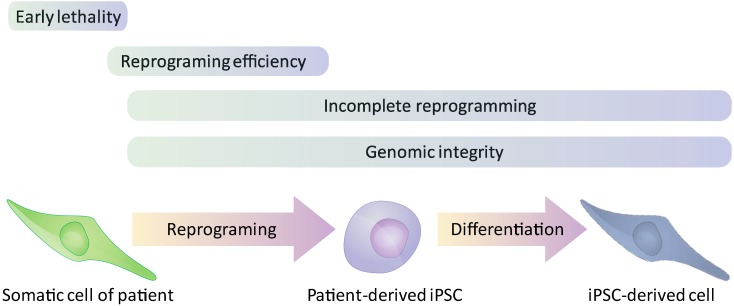
Limitations in the generation of iPSC-based disease models. The “X-axis” in this scheme depicts the specific stages during the formation of iPSC-based models that might be affected by the different factors that discussed in the main text.

Nevertheless, possible drawbacks in modeling genetic disorders by iPSCs suggest that some disorders or specific aspects within a given disease might be better modeled in ESCs than iPSCs. The generation of a faithful iPSC-based model might be disrupted due to the following reasons (see Figure 2): (1) Incomplete reprogramming as a result of “Epigenetic memory” of the original somatic cells [21,22,23]; (2) Mutations accumulated during the reprogramming process [24] and deleterious effects (such as chromosomal instability, [25]) of the reprogramming process on the genome integrity of iPSCs; (3) Genetic aberrations that significantly decrease the reprogramming efficiency [26]; (4) The absence of appropriate sources of somatic cells such as in the cases of genetic aberration and aneuploidies that lead to very early embryonic lethality [6].

To demonstrate the commonalities and differences between ESC- and iPSC-based models, we compared models for X-linked, autosomal recessive and autosomal dominant disorders in which disease-related phenotypes were observed in both models (Table 1). As expected, in some cases the ESC-based models and the iPSC-based models were similar (spinal muscular atrophy [27,28], Shwachman-Dimond syndrome [29], long QT syndrome [30], and some aspects of myotonic dystrophy [31,32]). However, in other cases the iPSCs were limited in their capacity to model the disorder or specific aspects within the disorders. To demonstrate some of these cases, and to discuss the principles behind them, we will focus on the following disorders: Turner syndrome, Fanconi Anemia, fragile X syndrome and Huntington’s disease.

## 3. Turner Syndrome

X chromosome monosomy (XO) is one of the most common chromosomal abnormalities, as 3% of all pregnancies start with XO embryos [33]. Yet, approximately 99% of the XO embryos undergo miscarriage during the first trimester [33,34]. The 1% that survive to term are born with Turner syndrome which is characterized by several phenotypes; the most common among them are growth failure, gonadal dysgensis and webbed neck [34]. While Turner syndrome derived iPSCs can be used in order to study the phenotypes of the patient (pending the availability of the required differentiation protocols), they might be problematic in modeling the early lethality of XO embryos, as they represent the exceptional 1% of the cases that survived to term.

In agreement with this notion, gene expression analysis of XO ESCs (derived by screening for ESCs with normal karyotype that lost one of their X chromosomes) revealed a significant effect of X chromosome monosomy on the expression of placental genes and suggests that the reason for the early lethality is abnormal placental development [6]. By contrast, there was almost no effect of X chromosome monosomy on placental gene expression in iPSCs derived from Turner syndrome patients, and even from amniotes of a 20 weeks old embryo [35]. The results suggest that Turner syndrome iPSCs represent the rare cases in which the embryo survived despite the XO karyotype.

**Table 1:** Comparison between ESCs and iPSC models for human genetic disorders.

Disease		ESCs		iPSCs		ESCs *vs.* iPSCs
		Reference (ref #)	Method of Derivation of Mutate ESCs	Reference (ref #)	Reprogramming Method	
**X-linked**	Fragile X	Eiges 2007 [7]	PGD	Urbach 2009 [36]	Retroviruses	**ESCs > iPSCs** In iPSCs *FMR1* is already methylated and inactivated (due to an epigenetic memory), thus iPSCs can’t be used to study the molecular mechanism related to *FMR1* gene silencing in Fragile X
						**ESCs < iPSCs** In iPSCs the *FMR1* gene is already inactivated, therefore iPSCs are a better system to study the effect of the gene silencing on neuronal development
	Rett syndrome			Cheung 2011 [37]	Retroviruses	**ESCs > iPSCs** Normal Female patients are heterogeneous in regard to the expression of *MECP2* (due to random X inactivation) but the iPSCs are homogeneous population of mutant cells (due to epigenetic memory of the inactivated X chromosome)
		Li 2013 [38]	Gene targeting by TALEN	Marchetto 2010 [39]	Retroviruses	**ESCs ~ iPSCs** iPSCs are heterogeneous population (Random X inactivation). Different phenotype has been examined in ESCs and iPSCs The discrepancy between the two iPSCs models illustrated the complexity of X inactivation in reprogramming
	Turner syndrome	Urbach 2011 [6]	Screen for XO colonies	Li 2012 [35]	Retroviruses and Lentiviruses	**ESCs > iPSCs** iPSCs represents the exceptional 1% of patients that survived to term and therefor can’t be used in order to study the effect of X chromosome loss on early lethality
**Autosomal recessive**	Fanconi Anemia	Yung 2013 [25]; Tulpule 2010 [40]	Knockdown of FANCA and FANCD2	Yung 2013 [25]	Lentiviruses	**ESCs > iPSCs** Very low reprogramming efficiency. The mutated iPSCs have many chromosomal aberrations and didn’t give rise to normal teratoma. Both ESCs and iPSCs showed hematopoietic phenotypes related to FA
		Liu 2014 [41]	Gene targeting by TALEN	Liu 2014 [41]	Reprogramming	**ESCs ~ iPSCs** Very low reprogramming efficiency, however, iPSCs have normal karyotype and normal characterization of pluripotent cells. Both ESCs and iPSCs demonstrated FA related phenotypes
	Spinal muscular atrophy	Wang 2013 [27]	Knockdown of SMN	Ebert 2009 [28]	Lentiviruses	**ESCs ~ iPSCs** Different phenotypes has been examined in ESCs and iPSCs Both models demonstrated abnormal motor neuron phenotypes
	Shwachman-Diamond syndrome	Tulpule 2013 [29]	Knockdown of SBDS	Tulpule 2013 [29]	Retroviruses	**ESCs ~ iPSCs** Both models demonstrated SDS related phenotype
**Autosomal dominant**	Long QT	Bellin 2013 [30]	Gene targeting	Bellin 2013 [30]	Retroviruses	**ESCs ~ iPSCs** Both models demonstrated Long QT related phenotypes. ESCs derived cardiomayocytes were electrophysiologically less mature than those derived from the iPSCs. Could be explained due to inherent variability between specific iPSCs and ESCs lines
	Huntington’s disease	Lu 2013 [42]	Over-expression of HTTexon1 with 23, 73 or 145 glutamine repeats in HESCs	HD iPSC Consortium 2012 [43]	Lentiviruses (OSKM + Nanog + Lin28)	**ESCs > iPSCs** (Based on Lu *et al.*) mHTT aggregated appeared only in ESCs based model
	Myotonic Dystrophy	Seriola 2011 [31]		Du 2013 [32]	Retroviruses	**ESCs ~ iPSCs** TNR becomes stable upon differentiation in ES and iPS. Down-regulation of MMR genes upon differentiation was observed only in ESCs

Representative examples of ESC and iPSC models for X linked, autosomal recessive and autosomal dominant disorders. As the primary intention of this review is to discuss the differences between the two model systems and to highlight cases in which the ESCs model is a better choice than the iPSCs model, only the characteristics or phenotypes that are relevance for the direct comparison between the two models were mentioned in the table. We refer the readers to the original papers to learn more details about each model. When several studies for a specific disorder were relevant for the comparison between the systems we cited all relevant studies.

## 4. Fanconi Anemia

Fanconi Anemia (FA) is an autosomal recessive disorder caused by a mutation in any of the 16 *FANC* genes and characterized by congenital abnormalities, cancer predisposition and progressive bone marrow failure [44]. Initial attempts to reprogram somatic cells from FA patients into iPSCs failed unless using fibroblasts that were first genetically corrected [45]. These results suggested that the FA pathway is essential for the reprogramming pathway (probably due to defective DNA repair and genomic instability of FA cells) and therefore that FA can’t be easily modeled by iPSCs. However, further attempts to reprogram “uncorrected” somatic cells from FA patients under hypoxic conditions [26], and even under normoxic conditions [25], showed that iPSCs can be derived from FA somatic cells, albeit in a very low efficiency and revealed that “…somatic cells harboring mutations that render the FA pathway defective are resistant but not refractory to reprogramming” [26]. Nevertheless, significant chromosomal aberration in uncorrected FA-iPSCs [25], but not in FA-iPSCs derived from “corrected” somatic cells [26] or in human ESCs with stable knockdown of FANCC [25] suggests that the FA pathway is required to prevent DNA damage and chromosomal instabilities associated with the reprogramming process. The severe aneuploidy in the uncorrected FA-iPSCs but not in the ESC-based model for Fanconi anemia suggests that ESCs and not iPSCs should be used to study FA. Surprisingly though, it has been recently shown [41] that FA-iPSCs with a normal karyotype can be derived from FA somatic cells upon episomal reprogramming. Moreover, the FA-iPSCs were very similar to FA-ESCs that were generated by gene targeting of the *FANCA* gene using the TALEN mediated gene targeting [37]. The FA-iPSCs and the FA-ESCs were extensively studied and compared to isogenic control cells (the original ESCs and target corrected FA-iPSCs) and proved to be a very useful model for different aspects of Fanconi anemia. While the reasons for the differences in the chromosomal stability between the viruses mediated reprogramming and the integration-free episomal mediated reprogramming are still not clear, these results indicate that in some cases, the reprogramming method itself might have a dramatic effect on the quality of the iPSCs and thus, should be taken under consideration when choosing to generate a disease model by reprogramming of somatic cells from patients.

## 5. Fragile X Syndrome

Fragile X syndrome (FXS) is a trinucleotide repeat disorder and is the leading cause of inherited intellectual disability in males, affecting approximately one in every four thousand boys and one in eight thousand girls worldwide [46,47,48,49]. The mutation leading to the syndrome is a trinucleotide CGG expansion at the 5′ untranslated region of the fragile X mental retardation 1 (*FMR1*) gene, which is accompanied by epigenetic changes, resulting in the silencing of the gene [49,50]. The product of the *FMR1* gene is the fragile X mental retardation protein (FMRP) which is most abundant in the brain and testis and plays a major role in synaptic plasticity [51].

In 2007 human ESCs from FXS affected embryos (FXS-ESCs) were derived for the first time through PGD and enabled the study of the development of the disease [7]*.* Interestingly, although carrying the full mutation, FXS-ESCs showed both *FMR1* mRNA expression and the presence of FMRP. This finding showed that the transcriptional silencing of *FMR1* is a developmentally regulated process. Moreover, the study indicated that *FMR1* is silenced in FXS embryos only during development and that the inactivation is initiated by chromatin modifications prior to DNA methylation [7]. Other studies on FXS-ESCs supported the finding that *FMR1* is expressed in full mutation embryos and is silenced only during differentiation and further demonstrated that *FMR1* plays an important role in early stages of neurogenesis and synaptic function [52,53]. Therefore, FX-ESCs are invaluable to study many aspects of FXS, first and foremost the epigenetic silencing mechanism. However, there are also limitations in the FX-ESCs model: FXS is represented by profound variability in patients, ranging from the varying length of the repeats, through the methylation levels, and to the neurological phenotype itself. The degree of intellectual impairment also varies between different individuals, as only about 30% of full mutation carriers display autistic behavior [54,55]. Additionally, some carriers of the full mutation allele do not display any of the syndrome’s phenotypes [56,57]. As this variability is not inherited from the parents and is detected only after PGD analysis, the probability of acquiring numerous human embryonic stem cells displaying the entire spectrum of genetic and epigenetic differences is quite small and may take several years.

In contrast to the *FMR1* expression seen in FXS-ESCs, it seems that in FXS derived iPSCs (FXS-iPSCs), despite successful reprogramming of patients derived fibroblasts, the *FMR1* gene is resistant to the process and remains methylated and silent [36,58,59]. Thus, while the FXS model in human ESCs demonstrated the temporal silencing of *FMR1*, in FXS-iPSCs *FMR1* was already inactive in the undifferentiated state. This fundamental difference between FXS-ESCs and FXS-iPSCs controls the choice of model according to the question being asked. In order to better understand the different aspects of the initiating steps of the *FMR1* silencing such as CGG methylation and the epigenetic silencing, one should use the FXS-ESC model. On the other hand, if one wishes to model neural development, screen for new drugs or understand the CGG expansion mechanism it is preferential to use the FXS-iPSC model to understand the effects of lack of FMRP on developing neurons, as we do not fully understand at which time point during the differentiation process *FMR1* is silenced in ESCs *in vitro*. One example using FX-iPSCs to model Fragile X syndrome is a study aimed to evaluate the reactivation of *FMR1* in FXS-iPSCs and their neuronal derivatives through epigenetic modulation drugs. This study showed not only that reactivation is possible but also uncovered additional layers of epigenetic control on *FMR1* [60].

## 6. Huntington’s Disease

Huntington’s disease (HD) is an autosomal dominant neurological disorder caused by a trinucleotide repeat expansion and characterized by a late onset progressive neurodegeneration ending with death [61,62]. In HD, an expansion of a CAG repeat in the first exon of the Huntingtin (*HTT*) gene leads to a toxic gain of function activity of the mutant Huntingtin protein (mHTT), containing an increased number of polyglutamines at the *N* terminus [62]. These polyglutamine tails are then cleaved and accumulate as aggregates in the nuclei of neurons [63].

During the past few years, several groups have successfully created iPSC models for HD (HD-iPSCs) [11,43,64,65]. Some have further differentiated HD-iPSCs to neurons and showed increased caspase activity of neural precursors upon growth factor deprivation [64] or increased lysosomal activity in both HD-iPSCs and derived neurons [65]. The most comprehensive work done with the HD-iPSCs model system was performed by the HD-iPSC Consortium, in which several HD-iPSC lines were created and analyzed by a group of different labs [43]. In this work, HD-iPSCs were also differentiated into neural stem cells (NSCs) and neurons. HD-derived NSCs showed differential gene expression accompanied with changes at the protein level as well. Other changes observed were compromised energy metabolism, inability to fire action potential and increased cell death. Neurons also display increased death under different stress conditions most notably in lines containing longer repeats.

HD-iPSCs provide a useful model, however, it was never shown that they accumulate any insoluble aggregates, and thus cannot be used to study the formation and pathological contribution of this aggregates to the development of the disease. In order to study this aspect of the syndrome, normal ESCs were genetically engineered to express the polyglutamine repeats [42]. Neurons derived from these HD-ESCs matured over a period of several months and showed the polyglutamine aggregates. Similar to HD-iPSCs, HD-ESCs derived neurons exhibited progressive death under stress conditions. It was also shown using this model that reduction of mHTT by just 10% is sufficient to prevent toxicity and lowering the expression levels of *HTT* by up to 90% had no effect on neurons, opening the possibility to screen for new drugs to control the levels of mHTT. Thus, HD-ESCs may provide a stronger tool than HD-iPSCs in our understanding of the initiation and progression of the pathology of HD. However, work done on ESCs derived directly from an embryo with HD did not show the formation of polyglutamine aggregates [66]. Furthermore, HD embryos from PGD are not readily available, and due to the fact that HD is a late onset disease, we do not know the ultimate phenotype of these never developed embryos. In this case, more work should be done on both HD-ESCs and HD-iPSCs in an attempt to obtain more of the molecular phenotypes characteristic of the disease to create a better model system.

## 7. Disease Modeling by Gene Targeting of hPSCs

As mentioned above, hPSCs based models can be generated by the derivation of ESCs from affected embryos diagnosed by PGD or by genetic manipulation of normal hPSC cells. Down-regulation or over-expression of specific genes can be easily achieved by RNAi technologies (for down regulation) or by introduction of exogenous genes into the genome (for over-expression). While these methods proved to be very informative in some cases, they can’t mimic the natural occurring mutation in the patients and therefore the relevance of the finding to the disease might be questionable in other cases. To overcome this problem, one has to induce a specific mutation that is identical to the mutation occurring in patients. However, until lately, genome editing in mammalian cells was an extremely inefficient process [17], and therefore it was challenging to generate homozygous mutations in human cells using the traditional methods for gene targeting. The development of new technologies for gene targeting, (reviewed in details in [17]) especially the TALEN technology and the Cas/CRISPER technology have dramatically increased the efficiency of gene targeting in mammalian cells and enabled to correct specific mutations or to obtained homozygous mutations in reasonable efficiency in human pluripotent stem cells.

These methods enable, for the first time, the comparison between isogenic cells that differ only in the specific mutation under investigation. This can be achieved by induction of a specific mutation in otherwise normal ESCs, by correction of a specific mutation in iPSCs or by combination of both methods [30,41] One possible drawback in these methods is the possible off-target effect that might result in additional unplanned genetic aberrations [17]. To overcome this possibility it is important to design the targeting sequence in a way that will decrease the off-target effects and to target different sequences of the same gene. Among these methods, the CRISPR technique will probably become the first choice for most labs due to the combination of accuracy, efficiency and accessibility.

## 8. “Guidelines” for Choosing the Optimal Model System for a Given Disease

**Figure 3:**
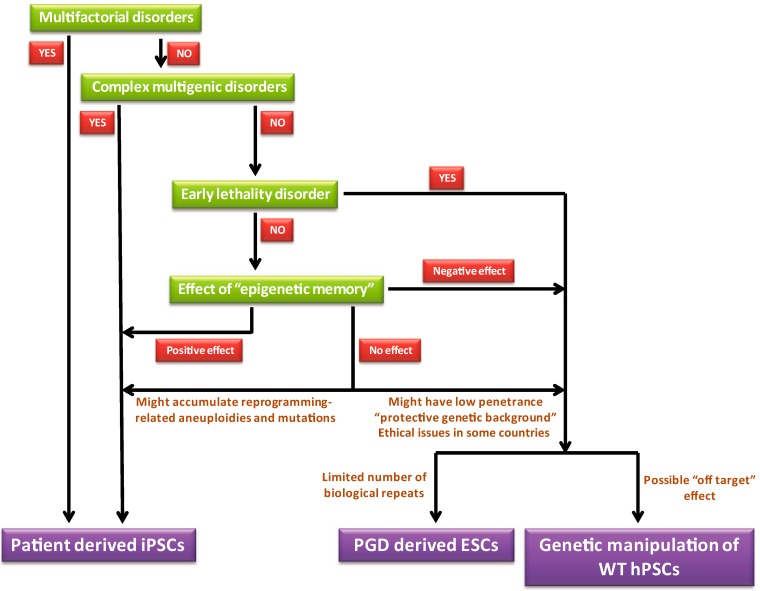
Scheme depicting the steps in choosing the appropriate system for disease modeling. While in some cases there is only one possible option (either ESCs or iPSCs), in other cases both ESCs or iPSCs can be used and the decision between the two methods should be done after the consideration of the advantageous and disadvantageous of each one of the options (some of them are described in the scheme).

The choice between modeling disorders with ESCs or iPSCs is dependent on several factors. We propose that the optimal model that probably overcomes most if not all the drawbacks mentioned above, is a model that combines both ESCs (that were genetically modified to carry a specific mutation) and iPSCs from patients (with an isogenic control of iPSCs from the same patient in which the mutation was corrected by genomic engineering). Such “combined methods” have been recently generated for long QT syndrome [30] and for Fanconi anemia [41]. However, as was discussed above, in some cases only one of the two methods is doable/informative. In Figure 3 we suggest general guidelines that should assist in choosing the right system for a given disorder. We generated this scheme based on the following assumptions:

Multifactorial disorders in which there is a major contribution to factors other than genetic factors on the disease etiology can be modeled exclusively by iPSCs from patients that already manifested the disease and can’t be modeled by ESCs. Similarly, iPSCs but not ESCs should be used to model multigenic disorders in which the genetic factor cannot be narrowed down into a single gene, (the discussion regarding the use of hPSCs to model these type of disorders is out of the scope of this review). Other than these two groups of disorders, “monogeneic” disorders can be modeled theoretically using both systems but under the following notions: (1) Genetic aberrations that lead to early lethality should not be modeled by iPSCs that derived from individuals that survived to term as they represent the exceptional cases (these rare cases however, can be used to study the effect of the genetic aberration on the phenotype of the exceptional embryos that survived to term and the genetic backgrounds that enable them to escape the early lethality); (2) Possible epigenetic memory in iPSCs has to be taken under consideration. While in general the epigenetic memory is considered to have a negative effect on iPSCs (as the pluripotent cells retain some of their previous identity of adult cells and therefore might not be equivalent to normal pluripotent cells), one can also utilize these phenomena in a positive manner. For example, in cases of hematopoietic disorders, iPSCs that were derived from blood cells might undergo hematopoietic differentiation in a greater efficiency than ESCs or iPSCs derived from other somatic cells [16]; (3) In cases in which no epigenetic effect is predicted, the best choice is to combine both model systems. When only one of the two systems will be used it is important to keep in mind the following limitations of each one of the methods: (a) The reprogramming process itself might results in accumulation of genetic aberrations that under some circumstances might affect the reliability of the model; (b) The penetrance of the mutation in the ESCs based model might not be completed (as a result of “protective” genetic background). By contrast, iPSCs are derived from patients that already manifested the phenotype and therefore one should not be concerned about “protective” genetic background; (c) In the case of PGD-based models the number of available samples (affected embryos) might be limited; (d) Gene targeting by the TALEN or CRISPER systems might lead to off-target effects.

## 9. Conclusions

Reprogramming of somatic cells from patients is a relatively easy procedure that doesn’t involve the usage of human embryos, nor ethical issues, and results in the formation of iPSCs with the naturally occurring mutation. Therefore, since the first derivation of human iPSCs from normal donors [67,68,69] and from patients [11], this method was considered by many to be the optimal methodology for disease modeling by human pluripotent cells (due to scientific reasons as well as other reasons). Indeed, during the last several years numerous models for genetic disorders were generated by reprogramming of somatic cells from patients. Yet, in many cases the mutant cell lines were not further analyzed to study their relevance to the actual disorders. In this review we focused on iPSCs based models and ESCs based models that have been shown to have a phenotype related to the disease.

To demonstrate that iPSCs can’t always replace ESCs in disease modeling, we focused on four models, each one emphasizes a specific aspect of the differences between ESC-based models and iPSC-based models. In addition to these specific examples, the reprogramming process itself might result in the generation of *de-novo* mutations [24] that might add “noise” to the system. On the other hand one general advantage of iPSC-based models compared to ESCs-based models is the fact that the patient chosen already manifested the phenotype associated with the mutation. This assures that there is no effect to the specific genetic background on the penetrance of the mutation. Based on the comparison between ESC-based models and iPSC-based models we suggested in Figure 3 a general guidelines to assist in choosing the appropriate model for a given disorder.

Lastly, the development of the “iPSCs technology” by Takahashi and Yamanaka some eight years ago [70], dramatically changed the entire field of pluripotent stem cells biology. While the most desirable application of this technology is probably for cell therapy, there is no doubt that currently the most common application of iPSCs is for disease modeling. In this review we highlighted some of the pros and cons of iPSCs compared to ESCs in regards to disease modeling and discussed the effect of advanced technologies for genome editing on the field. We believe that the field of disease modeling by hPSCs has reached a point wherein the challenge is not to derive pluripotent cells (ESCs or iPSCs) with a specific mutation but rather to better understand the pathophysiology of the disease and finding effective therapies.
